# Systemic symptoms as markers for severity in sepsis

**DOI:** 10.1186/cc14086

**Published:** 2015-03-16

**Authors:** J Edman-Wallér, L Ljungström, R Andersson, G Jacobsson, M Werner

**Affiliations:** 1 Södra Älvsborg Hospital, Borås, Sweden; 2 Skaraborg Hospital, Skövde, Sweden; 3 Institute of Biomedicine, University of Gothenburg, Sweden

## Introduction

The objective of this study was to evaluate six general symptoms as markers for severe sepsis in patients with suspected bacterial infections. Severe sepsis is a common cause of death and morbidity. Early detection and treatment is critical for outcome. Clinical presentation varies widely and no single test is able to discriminate severe sepsis from uncomplicated infections or non-infectious emergencies. Apart from local symptoms of infection, the systemic inflammatory reaction itself may give rise to general symptoms such as muscle weakness and vomiting.

## Methods

We present an observational, consecutive study. Data from ambulance and hospital medical records were analyzed. The survey included 290 patients (mean age: 70.6 years; median age: 74 years; male: 47%) who were admitted to a 550-bed secondary care hospital, receiving intravenous antibiotics for suspected community-acquired infections. General symptoms (fever/shivering, dyspnea, muscle weakness, gastrointestinal symptoms, localized pain, altered mental status) that were part of the reason the patient sought medical care were registered. Additionally, age, sex, vital signs, C-reactive protein, and blood cultures were registered. Patients that within 48 hours from admission fulfilled any criteria for severe sepsis were compared with patients that did not. Odds ratios for severe sepsis were computed using univariable as well as multivariable logistic regression, controlling for age and gender as confounders.

## Results

Severe sepsis criteria were fulfilled in 31% (*n *= 91) of the patients. These were older (median age: 79 years vs. 71 years) and experienced more symptoms (mean: 2.2, SD 0.9 vs. mean: 1.4, SD 0.7) than patients without severe sepsis, while there was no difference in C-reactive protein levels (OR per 50 mg/l: 1.07, 95% CI: 0.96 to 1.20). Among individual symptoms, altered mental status (OR: 4.4, 95% CI: 2.2 to 9.0), dyspnea (OR: 3.5, 95% CI: 2.1 to 5.9), and muscle weakness (OR: 2.2, 95% CI: 1.0 to 4.4) were significantly related to severe sepsis. Adjusting for age and sex, altered mental status (adj. OR: 4.1, 95% CI: 2.0 to 8.4) and dyspnea (adj. OR: 3.1, 05% CI: 1.8 to 5.3) remained significant.

## Conclusion

General symptoms, especially altered mental status and dyspnea, appear to be more common in severe sepsis than in milder infections. These symptoms might be utilized as a diagnostic aid for severe sepsis in the clinical setting, complementing vital signs and laboratory tests.

